# Root physiological adaptations involved in enhancing P assimilation in mining and non-mining ecotypes of *Polygonum hydropiper* grown under organic P media

**DOI:** 10.3389/fpls.2015.00036

**Published:** 2015-02-04

**Authors:** Daihua Ye, Tingxuan Li, Zicheng Zheng, Xizhou Zhang, Guangdeng Chen, Haiying Yu

**Affiliations:** College of Resources and Environment, Sichuan Agricultural UniversityChengdu, China

**Keywords:** organic phosphorus (Po), phytate, root morphology, acid phosphatase, phytase, phytoremediation

## Abstract

It is important to seek out plant species, high in phosphorus (P) uptake, for phytoremediation of P-enriched environments with a large amount of organic P (Po). P assimilation characteristics and the related mechanisms of *Polygonum hydropiper* were investigated in hydroponic media containing various concentrations of Po (1–8 mmol L^-1^) supplied as phytate. The mining ecotype (ME) showed significantly higher biomass in both shoots and roots compared to the non-mining ecotype (NME) at 4, 6, and 8 m mol L^-1^. Shoot P content of both ecotypes increased up to 4 mmol L^-1^ while root P content increased continually up to 8 mmol L^-1^ for the ME and up to 6 mmol L^-1^ for the NME. Root P content of the ME exceeded 1% dry weight under 6 and 8 mmol L^-1^. The ME had significantly higher P accumulation in both shoots and roots compared to the NME supplied with 6 and 8 mmol L^-1^. The ME showed higher total root length, specific root length, root surface area, root volume, and displayed significantly greater root length, root surface area, and root volume of lateral roots compared to the NME grown in all Po treatments. Average diameter of lateral roots was 0.17–19 mm for the ME and 0.18–0.21 mm for the NME. Greater acid phosphatase and phytase activities were observed in the ME grown under different levels of Po relative to the NME. This indicated fine root morphology, enhanced acid phosphatase and phytase activities might be adaptations to high Po media. Results from this study establish that the ME of *P. hydropiper* is capable of assimilating P from Po media and is a potential material for phytoremediation of polluted area with high Po.

## INTRODUCTION

Soils contain an enormous amount of phosphorus (P), commonly the bulk (greater than 50%) of which is fixed into organic P (Po) depending on land use, location, and soil type (Kong et al., 2014; Nash et al., 2014). Orthophosphate monoesters constitute the majority (up to 90%) of the soil Po pool. Inositol phosphates belonging to orthophosphate monoesters, particularly phytate (inositol hexaphosphate), and its metal ion compounds, are the most abundant forms of soil Po (Turner et al., 2002; Condron et al., 2005). Most terrestrial plants are unable to assimilate Po (Starnes et al., 2008; Sharma and Sahi, 2011). To satisfy plant requirements for available P, substantial P-based fertilizers (e.g., chemical fertilizer and animal manure) are used in many agricultural production systems (Pant et al., 2004; Nash et al., 2014). Monogastric animals (e.g., poultry and swine) are unable to digest the phytate in animal feeds. Therefore, the excreted manure contains a large number of undigested feed phytate, which leads to redistribution of Po (Priya and Sahi, 2009) and accumulation of total P in soils. With the development of the livestock industry, some regions of China have problems with animal manure disposal and its negative impacts on water quality (e.g., eutrophication) due to P run off. Phytate has become a potential pollutant of waters (Maougal et al., 2014). In addition, inositol phosphates exist in large amounts in water environments, which may make contribution to eutrophication (Turner et al., 2002). Therefore, the potential environmental pollution caused by phytate arising from long-term and iterative applications of P-based fertilizers and arbitrary emission of animal manure should be addressed.

Phytoremediation is able to be used as an innovative tool to remove P from P-polluted soils or eutrophic water (Sharma et al., 2004). Some crops and grasses have been verified to be effective for phytoremediation in high P environment. Sharma et al. (2007) reported that several vegetable species of yellow squash (*Cucurbita pepo* var. *melopepo*) and slicing cucumber (*Cucumis sativus*) were screened as potential P accumulators with shoot *P* > 1% dry weight (DW). Marshall and Gulf ryegrass (*Lolium multiflorum* L.) could accumulate great shoot P (excess of 1% DW) from P-enriched nutrient solution, agar media or soils (Sharma et al., 2004; Sharma and Sahi, 2005, 2011). A hybrid grass (*Duo festulolium*) originated from the hybridization between a ryegrass (*L. perenne* L. or *L. multiflorum* L.) and Meadow Fescue (*Festuca pratensis* H.) was also identified as a P accumulator for phytoremediation (Priya and Sahi, 2009).

Plants are known to meet P requirements by utilizing phosphate anions. Commonly, Po compounds must be mineralized into inorganic P (Pi) to provide important P sources. Acid phosphatase (Apase) can catalyze the hydrolysis of Po compounds to release Pi for plant uptake (Scott and Condron, 2003). Phytase resulting from plant or microbial secretion can realize the mineralization of phytate to yield plant-available Pi (Sharma et al., 2007; Starnes et al., 2008). Root enzymes of APase and phytase have been shown to contribute to P uptake potentials, and they play vital roles involved in regulating P nutrition (Richardson et al., 2000; Ramesh et al., 2011; Ye et al., 2014b). Plants are able to develope fine root systems to improve P acquisition efficiency (Vance et al., 2003; Lambers et al., 2006). Some attempts have been made to proclaim that P assimilation is correlated with total root length, root surface area, lateral root length, and number, and specific root length when plants are grown in P-deficient conditions (Li et al., 2007; Hammond et al., 2009; Zhang et al., 2013a). However, little is known about the root morphological mechanisms involved in P assimilation for a P accumulator grown in P-sufficient conditions.

*Polygonum hydropiper*, including a mining ecotype (ME) and a non-mining ecotype (NME), is able to grow both on land and in water as a kind of annual herbage. *P. hydropiper* is generally used as a seasoning or a kind of herbal medicine with alexeteric and styptic effects (Ye et al., 2014a). The ME and the NME showed fine growth and great shoot P accumulation when plants were grown in hydroponic media and soils with high concentrations of Pi (Xiao et al., 2009; Huang et al., 2012; Ye et al., 2014b). Recently, P accumulation characteristics and P removal potentials in the two ecotypes of *P. hydropiper* have also been analyzed in livestock wastewater and swine manure amended soils (Zheng et al., 2013; Ye et al., 2014a). The above studies indicated that the ME demonstrated greater P accumulation than the NME when grown under high P media where Po existed and they laid the foundations for revealing a P-phytoremediation strategy in the two ecotypes of *P. hydropiper* using hydroponic media supplied with only Po. In this study, we hypothesized the growth and P uptake from a series of Po concentrations supplied as phytate would still present differences in the two ecotypes of *P. hydropiper*. The objective of our work was to ascertain the factors leading to the differences between ME and NME and identify if the ME was more suitable for phytoremediation of high-Po soils and waters. Thus, characteristics of P accumualtion, root morphology, activities of Apase and phytase were determined in the two ecotypes of *P. hydropiper*, and the roles of root physiological adaptations in the assimilation of P from phytate were investigated.

## MATERIALS AND METHODS

### COLLECTION OF SEEDLINGS

Seedlings of *P. hydropiper* including a ME and a NME were collected in May 2013. The NME was sampled from a non-contaminated agricultural area in Yucheng, Ya’an, Sichuan, China (102°59′ E, 29°59′ N) with average annual temperature of 16.1°C and average annual precipitation of 1732.0 mm. The ME was collected from a P-mine site in Shifang, Sichuan (104°50′ E, 30°25′ N) which has similar climatic and topographic environments. Healthy and uniform seedlings were chosen, the roots sterilized, and pre-cultured on vermiculite for 1 week based on the method of Huang et al. (2012).

### GROWTH OF SEEDLINGS

Healthy, similar sized seedlings (∼10 cm) were selected for the pot experiments. Modified Hoagland nutrient solution without monopotassium phosphate (containing 945 mg L^-1^ hydrated calcium nitrate, 506 mg L^-1^ potassium nitrate, 80 mg L^-1^ ammonium nitrate, 493 mg L^-1^ magnesium sulfate, 18.35 mg L^-1^ EDTA-Fe, 4.15 μg L^-1^ potassium iodide, 31 μg L^-1^ boric acid, 111.5 μg L^-1^ manganese sulfate, 43 μg L^-1^ zinc sulfate, 1.25 μg L^-1^ sodium molybdate, 0.125 μg L^-1^ cupric sulfate, and 0.125 μg L^-1^ cobalt dichloride) was used as basal nutrient medium for plant growth. To investigate the effect of different concentrations of Po, media were supplemented with 1, 2, 4, 6, and 8 mmol P L^-1^ added as phytate isolated from rice (*myo*-inositol hexaphosphoric acid dodecasodium salt, Sigma). The pH of these media was adjusted to 5.8 by 0.1 mol L^-1^ HCl or 0.1 mol L^-1^ NaOH during the culture period. The periphery of hydroponics container was covered with black paint to prevent light diffusion into the root. Pot experiments were carried out using barrels with volume of 5 L full of nutrient solution. Apertured, hard cystosepiment (thick of 2 cm, aperture of 2 cm and two apertures) was chosen as barrel cover. The cystosepiment and sponge were used to fix the seedlings to keep the roots in the barrels and maintain the shoots in the above of cystosepiments. Two uniform and healthy seedlings of *P. hydropiper* were planted in each barrel based on the principle of “one aperture, one seedling.” The aforementioned ‘two seedlings per barrel’ was just one replicate per treatment and four replicates were performed for each treatment. The nutrient media were replaced every 5 days. Pots were placed according to a completely randomized design in a greenhouse with sunlight.

### HARVEST AND ANALYSIS OF ROOT MORPHOLOGY

The plants were harvested after 5 weeks of growth. Samples harvested from different treatments were washed thoroughly with running tap water and distilled water, respectively. Fresh roots were cut from shoots, then positioned in a plexiglass tray full of water. These suspended roots were gently detangled using tweezers and distributed in the plexiglass tray. Subsequently, scanning the roots was conducted by Epson perfection V700 photo, Japan. Digital images of the whole root system were achieved by multiple segments per plant. These images were computerized and analyzed by digital imaging software WinRHIZO V2007d (Regent Instrument Inc., Canada). Root length, root surface area, and root volume of each plant were measured immediately. Parts of main and lateral roots in these images were randomly selected to measure their diameter. Base on the real diameter of main and lateral roots, threshold of 0.4 mm was able to well differentiate the two kinds of roots. Thus, two levels of >0.4 and ≤0.4 mm were used to divide roots into main roots and lateral roots. Root length, root surface area, root volume, and average diameter for main and lateral roots were also determined. Specific root length was estimated based on the method of Pang et al. (2010), namely, the root length per unit root DW. Tissues including aboveground parts (shoots) and underground parts (roots) were separately oven-dried at 80°C for 2 days to gain constant weight after imaging. Finally, the DW was recorded.

### ANALYSIS OF P CONTENT

To determine plant P content, the dried samples were ground with a stainless steel grinder (FW-100, Tianjin Taisite Instruments, China) and sieved through a 0.15 mm nylon sieve. 0.3 g ground samples were dissolved with 8 mL concentrated sulphuric acid in a 50 mL triangular flask using a hotplate at low temperature for 8 min. During this process, 0.5 mL of 30% hydrogen peroxide was added into the digested samples when they were cooled to room temperature. The procedure was repeated twice or three times till the samples were clear. Ultimately, the samples were heated at low temperature for 5–10 min to remove residual hydrogen peroxide. Tissue P content was determined colorimetrically at 700 nm by using an ultraviolet-visible spectrophotometer (Lu, 1999).

Commonly, the bioaccumulation coefficient (BCF) is used to estimate the enrichment capacity. The translocation factor (TF) is described to evaluate the translocation efficiency. Therefore, the BCF and TF were calculated based on the methods of Liu et al. (2009): BCF = root P content/P concentration in medium; TF = shoot P content/root P content.

### DETERMINATION OF APASE AND PHYTASE ACTIVITIES

Fresh root materials were frozen in liquid nitrogen and stored at -80°C after being washed thoroughly with running tap water and distilled water, respectively. Fresh tissues (∼0.3 g) were homogenized in ice bath with quartz sand in 5 mL of 15 mM 2-morpholinoethanesulfonic acid, monohydrate (MES) buffer with pH of 5.5 (containing 0.5 mM CaCl_2_H_2_O, 1 mM EDTA). The mixture was centrifuged at 4°C for 10 min at 14,000 rpm. After centrifugation, the supernatants were collected to use for analysis of Apase and phytase activities.

These enzyme activities were performed using p-nitrophenyl phosphate disodium salt hexahydrate (pNPP) and *myo*-inositol hexaphosphoric acid dodecasodium salt (IHP) as substrate for APase activity and phytase activity. Determination of Apase and phytase activities was done according to the methods of earlier reports (Sharma and Sahi, 2005; Starnes et al., 2008). In the case of APase activity determination, 2 mL of 10 mM pNPP was added into 1 mL extracted samples, and the mixture was incubated at 37°C for 30 min. Subsequently, the reaction was terminated by the addition of 0.25 M NaOH with the volume of 2 mL. For analysis of phytase activity, following the addition of 15 mM MES assay buffer (pH 5.5) into 1 mL of enzyme extract, the reaction was initiated with 2 mM of the corresponding substrate added into the previous mixture (total volume of 4 mL). To terminate the reaction, 2 mL of ice-cold 20% (w/v) trichloroacetic acid was added after incubation at 37°C for 60 min. APase and phytase activities were measured spectrophotometrically using an ultraviolet-visible spectrophotometer (model UN-2600A, UNICO) at 412 and 882 nm, respectively. In the presence of the enzymes activities, the substrate pNPP was hydrolyzed into p-nitrophenol (pNP) and the substrate IHP was hydrolyzed into soluble Pi. Therefore, APase activity was expressed as pNP μg g^-1^ fresh weight (FW) min^-1^. Phytase activity was calculated as mU g^-1^ root FW, where 1 U releases 1 μmol of soluble Pi min^-1^ quantified by the molybdenum-blue method.

### STATISTICAL ANALYSIS

Data from 4 replicates were sorted out by Excel (Microsoft) software packages. Statistical analyses were performed using variance analysis by the DPS software package (Version 11.0). The least-significant difference was used to estimate differences among plants or treatments at significant level of *p* < 0.05. Graphical work was accomplished by Origin (Version 8.0).

## RESULTS

### BIOMASS OF P. HYDROPIPER

**FIGURE 1:**
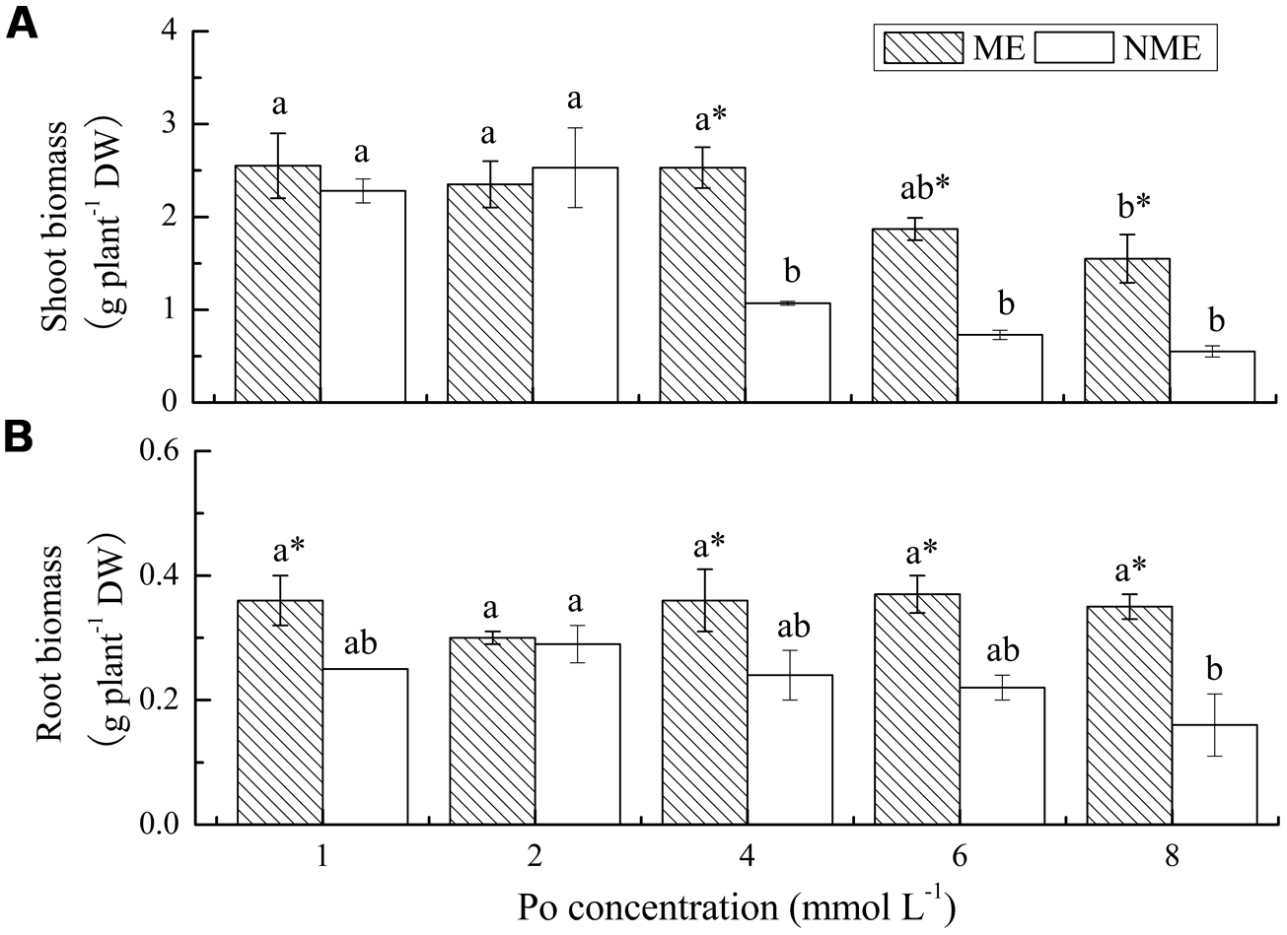
**Shoot biomass **(A)** and root biomass **(B)** of *Polygonum hydropiper* grown under hydroponic media containing various Po (1–8 mmol L^-**1**^) supplied as phytate for 5 weeks.** ME, mining ecotype, NME, non-mining ecotype. Values represent mean of four replicates ± SE. Small letters in each column are significantly different (*p* < 0.05) among Po treatments. *indicates significantly different (*p* < 0.05) between ecotypes.

Shoot and root biomass of the two ecotypes of *P. hydropiper* grown under hydroponic media containing various concentrations of Po up to 8 mmol L^-1^ are displayed in **Figure 1**. A significant decrease in shoot biomass was observed in media with 8 mmol L^-1^ for the ME and 4 mmol L^-1^ for the NME (**Figure 1A**). Growth in the ME was greater than in the NME at all concentrations of Po. In addition, shoot biomass of the ME was significantly higher relative to that of the NME when both were grown in media with 4, 6, and 8 mmol L^-1^. No significant difference in root biomass was noticed in the ME, whereas the NME exhibited a persistent decrease in various concentrations of Po (**Figure 1B**). Root biomass of the ME was significantly greater than that of the NME at any level of P except for 2 mmol L^-1^.

### P ASSIMILATION, TRANSLOCATION, AND ACCUMULATION

**FIGURE 2:**
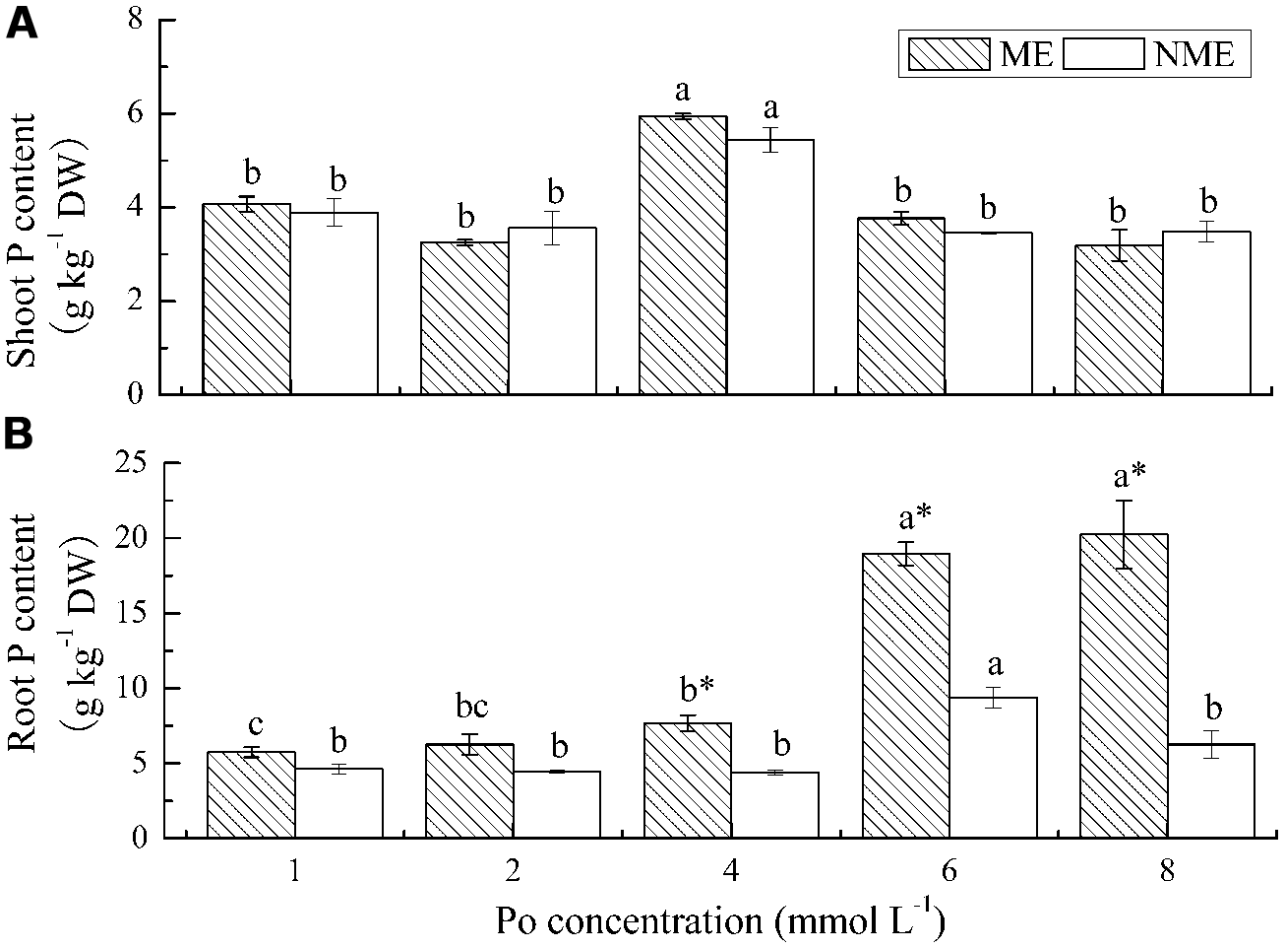
**Shoot P content **(A)** and root P content **(B)** of *P. hydropiper* grown under hydroponic media containing various Po (1–8 mmol L^-**1**^) supplied as phytate for 5 weeks.** ME, mining ecotype, NME, non-mining ecotype. Values represent mean of four replicates ± SE. Small letters in each column are significantly different (*p* < 0.05) among Po treatments. *indicates significantly different (*p* < 0.05) between ecotypes.

Shoot P content in both ecotypes increased with increasing applications of Po until it reached 4 mmol L^-1^ (**Figure 2A**). The maximum shoot P content was 5.94 g kg^-1^ DW for the ME and 5.44 g kg^-1^ DW for the NME. In the case of root P content, a persistence of sharp increase was observed in the ME with respect to the increasing Po levels in the growth media while a significant reduction occurred in the NME beyond 6 mmol L^-1^ (**Figure 2B**). Root P content in the ME was 5.74–20.24 g kg^-1^ DW, whereas root P content in the NME varied from 4.62 to 9.39 g kg^-1^ DW depending on Po concentration in the media. Root P content of the ME was 1.74, 2.02, and 3.23 times greater compared with that of the NME when grown in media supplied with 4, 6, and 8 mmol L^-1^, respectively.

As shown in **Table 1**, BCF in the ME of *P. hydropiper* was 1.24–3.23 times higher compared to that of the NME when grown in the same concentration media. TF of the ME was lower than that of the NME and TF for both ecotypes was lower than 1.0, suggesting that P mainly accumulated in the roots.

**Table 1:** Bioaccumulation coefficient and translocation factor of *Polygonum hydropiper* grown under hydroponic media containing various Po (1–8 mmol L^-**1**^) supplied as phytate for 5 weeks.

Po concentration (mmol L^-1^)	Bioaccumulation coefficient			Translocation factor	
	ME	NME		ME	NME
1	185.08	149.03		0.71	0.84
2	101.00	71.57		0.52	0.80
4	61.92	35.54		0.77	1.23
6	101.88	50.47		0.20	0.37
8	81.60	25.24		0.16	0.56

*ME, mining ecotype; NME, non-mining ecotype.*

**FIGURE 3:**
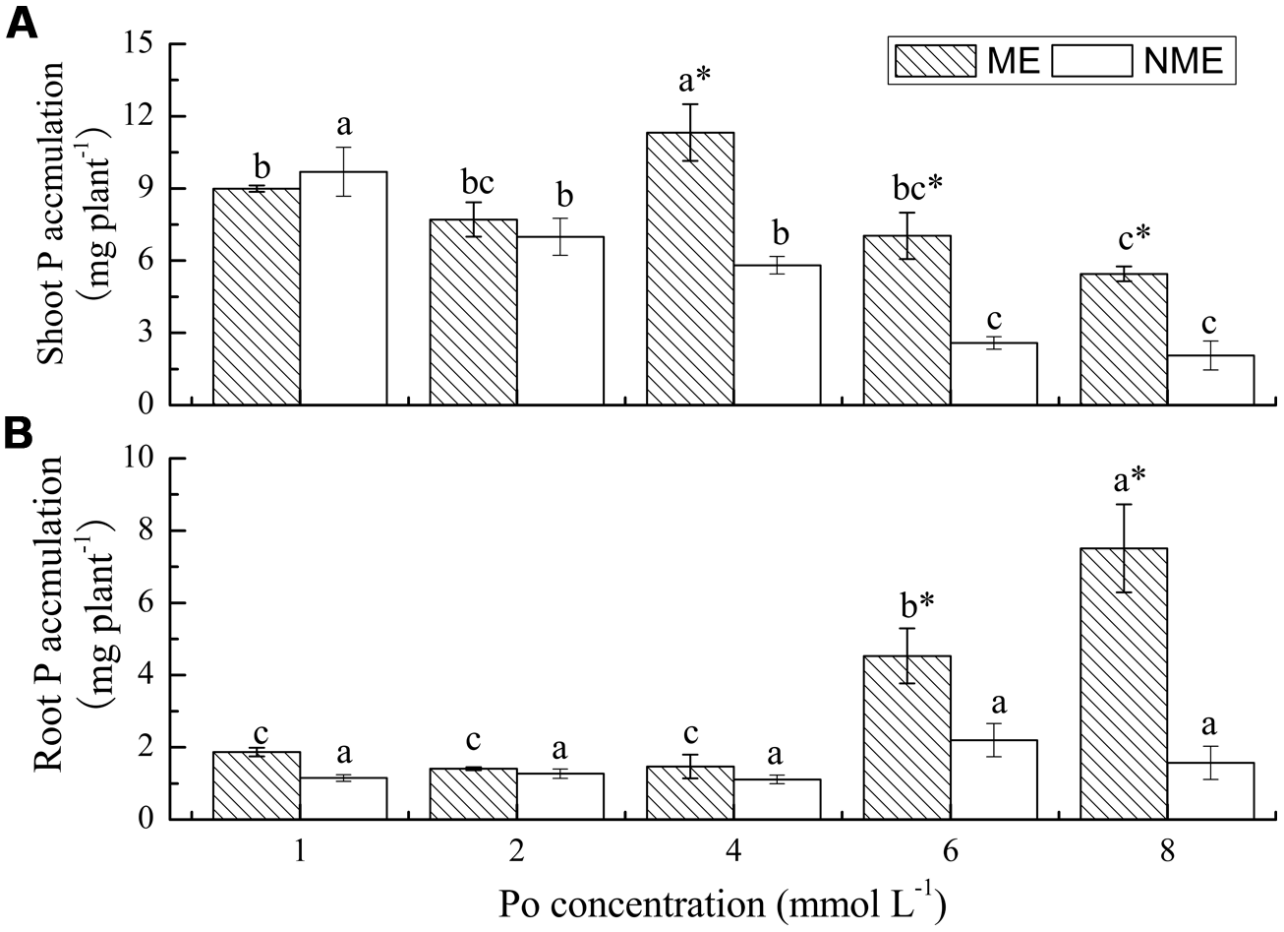
**Shoot P accumulation **(A)** and root P accumulation **(B)** of *P. hydropiper* grown under hydroponic media containing various Po (1–8 mmol L^-**1**^) supplied as phytate for 5 weeks.** ME, mining ecotype; NME, non-mining ecotype. Values represent mean of four replicates ± SE. Small letters in each column are significantly different (*p* < 0.05) among Po treatments. *indicates significantly different (*p* < 0.05) between ecotypes.

P accumulation is the product of biomass and P content. P accumulation in both shoots and roots of *P. hydropiper* were highly variable, depending on plant ecotypes and concentrations of Po in the growth media (**Figure 3**). Shoot P accumulation of the ME reached its peak value (11.32 mg plant^-1^) when grown in media supplemented with 4 mmol L^-1^. However, a continued decrease was observed in P accumulation of the NME. Furthermore, the pattern in root P accumulation of *P. hydropiper* was similar to that in root biomass. Root P accumulation of the ME significantly increased with the increasing Po concentrations and it was 2.06 and 4.78 times higher than that of the NME at 6 and 8 mmol L^-1^, respectively.

### ROOT MORPHOLOGY PER PLANT

**FIGURE 4:**
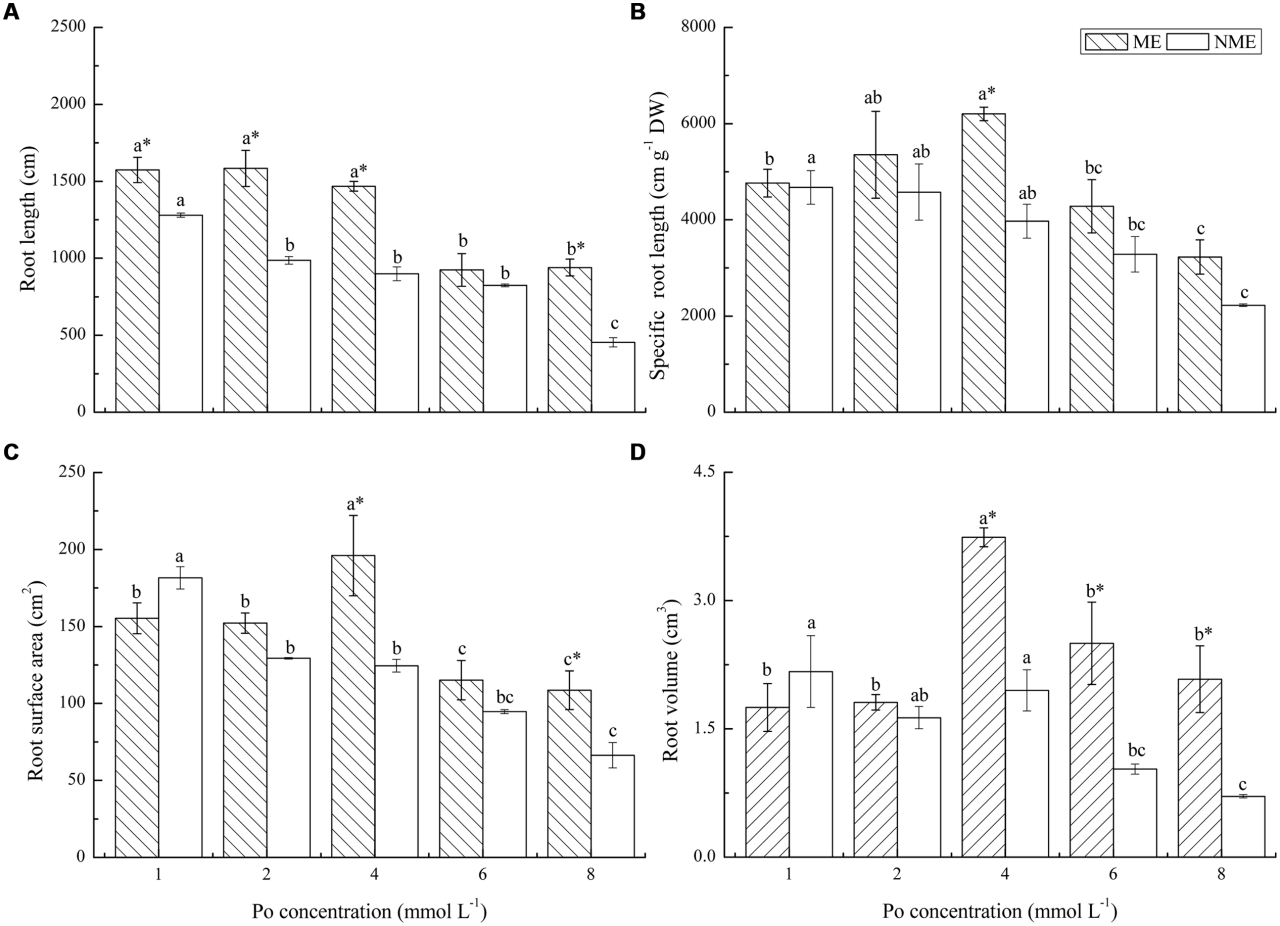
**Root system morphology: root length **(A)**; specific root length **(B**); root surface area **(C)**; root volume **(D)** of *P. hydropiper* grown under hydroponic media containing various Po (1–8 mmol L^-1^) supplied as phytate for 5 weeks.** ME, mining ecotype; NME, non-mining ecotype. Values represent mean of four replicates ± SE. Small letters in each column are significantly different (*p* < 0.05) among Po treatments. *indicates significantly different (*p* < 0.05) between ecotypes.

Morphological developments in the root systems of the two ecotypes subjected to various Po levels in the nutrient media are depicted in **Figure 4**. Root length, specific root length, root surface area, and root volume were determined in the study. Root length evidently decreased with Po concentration up to 6 mmol L^-1^ for the ME and 2 mmol L^-1^ for the NME, and the ME demonstrated larger root length in a range of 9.24–15.84 m compared to the NME. Specific root length of the ME reached its maximum (6201.48 m g^-1^ DW) at 4 mmol L^-1^, whereas in the NME specific root length continuously decreased in response to the increased Po addition in the media. The two ecotypes differed greatly in root surface area and root volume. Root surface area of the ME increased until the Po concentration reached 4 mmol L^-1^ in the nutrient media. Root surface area of the NME had a great sensitivity to Po application, with root surface area becoming less than that cultivated in the media with a relatively low Po concentration (1 mmol L^-1^). Root volume had the same tendency as root surface area in both ecotypes of *P. hydropiper.* The ME registered great root volume in rang of 1.75–3.74 cm^3^ while the NME showed a lower root volume ranging from 0.71 to 2.84 cm^3^.

### ROOT MORPHOLOGICAL PARAMETERS OF MAIN AND LATERAL ROOTS

Root length, surface area, volume, average diameter in main and lateral roots mainly affected by Po concentrations and ecotypes (**Table 2**). Main and lateral root length, surface area and volume of the NME decreases proportionally to the increase of Po. However, main root length of the ME increased with Po until a level of 4 mmol L^-1^, and the same phenomenon was observed in main and lateral root surface area and volume. The lateral root length of the ME is not affected at all until 4 mmol L^-1^. At levels greater than 4 mmol L^-1^, main and lateral root length, surface area and volume of the ME sharply decreases. Average diameter of main and lateral roots was less affected by Po concentrations compared to ecotypes. The ME demonstrated significantly greater lateral root length, surface area compared to the NME in each Po treatment. Furthermore, the ME displayed higher main root length, surface area compared to the NME when grown in 2 and 4 mmol L^-1^. Higher main and lateral root volume was observed in the ME than the NME at relatively high Po concentrations, viz. 4, 6, and 8 mmol L^-1^. In addition, lower average diameter of lateral roots in the ME was noticed relative to the NME.

**Table 2:** Root length, surface area, volume, average diameter of main roots, and lateral roots in *P. hydropiper* grown under hydroponic media containing various Po (1–8 mmol L^-**1**^) supplied as phytate for 5 weeks.

Po concentration	Ecotypes	Root length (cm plant^-1^)			Root surface area (cm^2^ plant^-1^)			Root volume (cm^3^ plant^-1^)			Average root diameter (mm)	
mmol L^-1^		Main roots	Lateral roots		Main roots	Lateral roots		Main roots	Lateral roots		Main roots	Lateral roots
1	ME	321.38bc	1361.95a*		83.02bc	77.54ab*		1.96b	0.45ab		0.74ab	0.18a
	NME	335.08a	894.48a		90.32a	59.28a		2.13a	0.39a		0.74a	0.21a*
2	ME	361.80b*	1281.81a*		92.28b*	75.17ab*		2.07b	0.42abc		0.77a*	0.17a
	NME	267.34ab	751.92b		64.57b	49.69ab		1.40abc	0.31ab		0.69ab	0.20a*
4	ME	468.81a*	1338.74a*		131.88a*	84.98a*		3.41a*	0.54a*		0.71b	0.18a
	NME	285.74ab	666.68b		62.13b	41.90ab		1.53ab	0.27abc		0.71a	0.18b
6	ME	246.37c	734.77b*		64.00c	62.28bc*		2.20b*	0.38bc*		0.72b*	0.18a
	NME	204.90bc	531.63c		44.04bc	39.85b*		0.80bc	0.23bc		0.65b	0.21a*
8	ME	256.47c*	712.61b*		59.29c	48.74c*		1.77b*	0.31c*		0.71b*	0.19a
	NME	170.69c	358.49d		35.57c	21.53c		0.61c	0.13c		0.66b	0.20a

ME, mining ecotype; NME, non-mining ecotype. Values represent mean of four replicates. Small letters in each column are significantly different (*p* < 0.05) among Po treatments. *indicates significantly different (*p* < 0.05) between ecotypes.

### ACTIVITIES OF APASE AND PHYTASE

**FIGURE 5:**
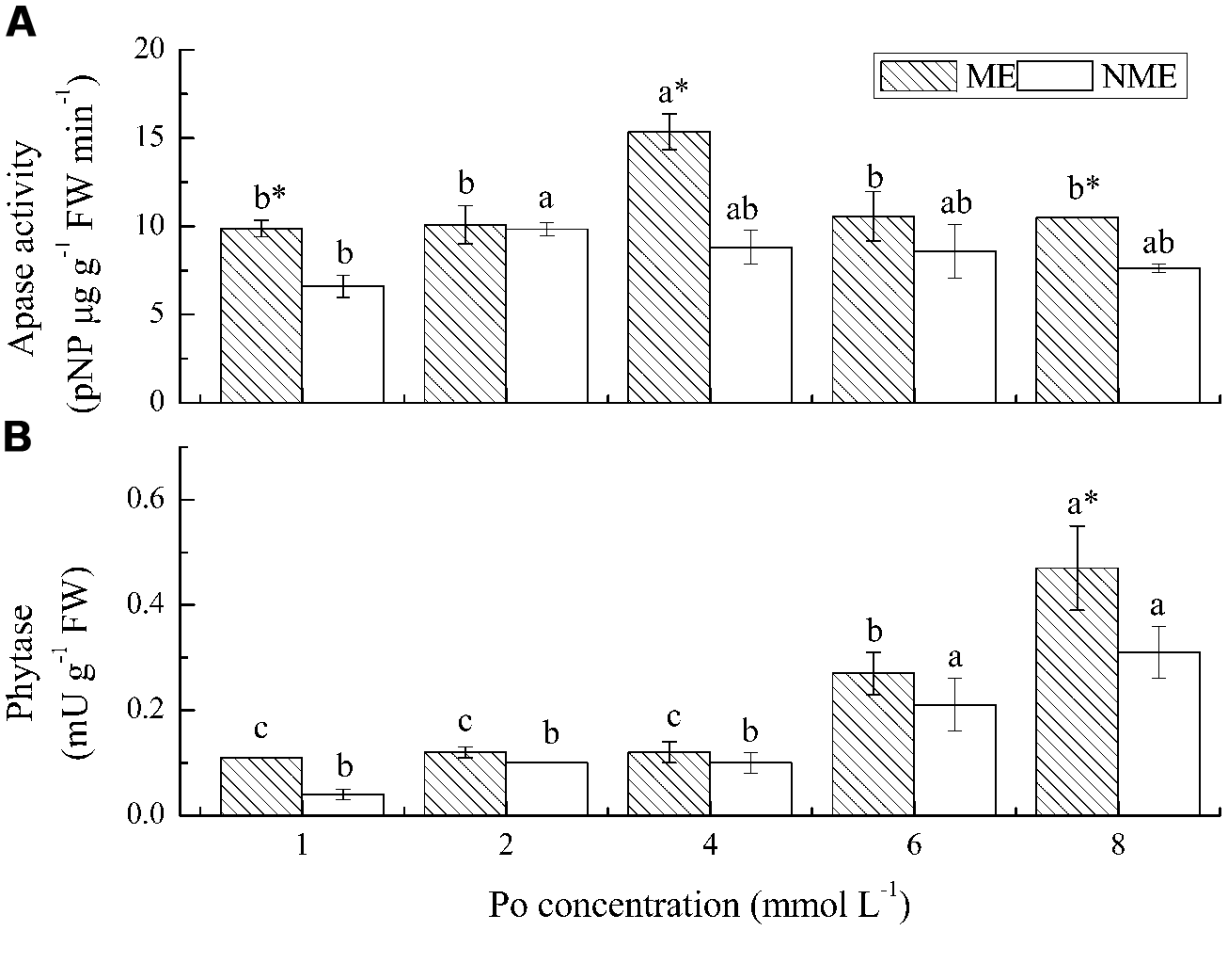
**Activities of APase **(A)** and phytase **(B)** in roots of *P. hydropiper* grown under hydroponic media containing various Po (1–8 mmol L^-**1**^) supplied as phytate for 5 weeks.** ME, mining ecotype; NME, non-mining ecotype. Values represent mean of four replicates ± SE. Small letters in each column are significantly different (*p* < 0.05) among Po treatments. *indicates significantly different (*p* < 0.05) between ecotypes.

The greatest increase in root APase activity were obtained from the ME grown under 4 mmol L^-1^ and from the NME grown under 2 mmol L^-1^ (**Figure 5A**). APase activities in the roots ranged from 9.87 to 15.37 pNP ug (g FW)^-1^ min^-1^ for the ME and from 6.61 to 9.83 pNP ug (g FW)^-1^ min^-1^ for the NME, respectively. Root APase of the ME displayed on average 30% higher relative to that of the NME. Phytase activity followed a different trend from APase in both ecotypes. Phytase activity of the ME increased in the growth media, irrespective of Po level, whereas that of the other ecotype had no significant increase (**Figure 5B**). Phytase activity in the ME was also higher (∼1.20–2.75-fold) than that in the NME grown in media containing various Po concentrations.

## DISCUSSION

### BIOMASS AND P ACCUMULATION OF P. HYDROPIPER

There are many toxicity symptoms in the plants including poor growth, smaller leaves, fewer branches, and even withered under high concentrations of Po. *P. hydropiper*, as a P-accumulator, was grown in the presence of high levels of Po added as phytate for 5 weeks (**Figure 1**). Fewer branches and smaller leaves were observed in the NME compared to the ME. The high Po reduced shoot growth in the ME but to a lesser extent compared to the NME. The data showed that the ME exihibited better adaptability, tolerance and less toxicity compared to the NME grown in phytate-rich media. Biomass in both shoots and roots of the ME was significantly greater than that of the NME under relatively high levels of Po (4, 6, and 8 mmol L^-1^). These results corresponded well with earlier researches which reported that the ME demonstrated greater biomass compared to the NME when grown in high P conditions (Huang et al., 2012; Ye et al., 2014a). Some scholars have indicated that many plants have limited ability to uptake and utilize P from phytate. Previous researches reported that wild-type plants of *Arabidopsis* (Richardson et al., 2001), *Glycine max* (Kong et al., 2014), and *Phaseolus vulgaris* (Maougal et al., 2014) showed significantly reduced biomass when grown under phytate media. Wheat plants (*Triticum aestivum* L.) grown in sterile phytate agar substrate, demonstrated no significant increase in shoot DW compared to without P-fed controls (Richardson et al., 2000). However, some scholars reported that Duo grass and annual ryegrass are able to obtain sufficient P from phytate to meet their P demands and registered better DW growth compared to the plants in the absence of P (Priya and Sahi, 2009; Sharma and Sahi, 2011).

Our previous study suggested that the ME was capable of regulating its internal P content through dilution of accumulation in DW or by not transferring P from root to shoot when the supplied P increased (Ye et al., 2014b). In this study, higher root P content than shoot (**Figure 2**) indicated not taking up the P into shoots may be an adaptation involved in reducing the P toxicity from phytate-rich media. The aerial part is taken to remove contaminant and root is much harder to collect from soils. However, the whole plant is relatively easy to harvest from eutrophication water. *P. hydropiper* can be grown in high P soil, as well as in water, providing various ways for high P remediation. The shoot P content of ME was exactly the same as NME (<1% DW) and significantly lower than Gulf and Marshall ryegrass grown in media containing various concentrations of phytate (Sharma and Sahi, 2011). However, P acquisition from phytate by the ME exceeded 1% DW in the roots. Therefore, this characteristic of ME is more suitable for purification of eutrophication water. Elephantgrass (*Pennisetum purpureum* Schum.) were verified to be available for P phytoremediation, however, its P content in the harvested shoot was just 2.9 g kg^-1^ (Silveira et al., 2013). Shoot P content in some aquatic plants, such as *Pistia stratiotes* and *Myriophyllum aquaticum* (Polomski et al., 2009), used for P remediation is also lower than that of the ME. So, not all plants used for phytoremediation demonstrated shoot P content as high as 1% DW. The success of phytoremediation is ultimately dependent on P quantities accumulated in the harvested parts (Pant et al., 2004). Even though great shoot P content was observed in several plants such as Duo grass and annual ryegrass, the total removed P is relatively inadequate due to their low DW yields. P accumulation in whole plant of the ME (9.12–12.79 mg plant^-1^) was steady in response to the increasing Po concentrations, whereas that of the NME (3.63–10.84 mg plant^-1^) significantly decreased under the same conditions. The data presented above were significantly higher compared to P accumulation of other plants grown under phytate agar media (Priya and Sahi, 2009; Sharma and Sahi, 2011), indicating that the ME of *P. hydropiper* is capable of utilizing phytate and it demonstrates great P uptake and removal potentials from Po-enriched water.

### ROOT PHYSIOLOGICAL ADAPTATIONS INVOLVED IN ENHANCING PO ASSIMILATION

Root physiological adaptations play important roles in enhancing P bioavailability (Shen et al., 2011). These adaptation mechanisms basically contain changes of root morphology to enhance spatial availability of P, organic acids secretion to mobilize less available Pi, and phosphatase or phytase exudation to mineralize Po (Zhang et al., 2010; Shen et al., 2011). P acquisition ability of plant is closely linked with root morphological parameters (Denton et al., 2006; Pang et al., 2010), and fine root morphology is propitious to maximize P assimilation. Root morphology parameters examined in our study contributing to P uptake efficiency included root length, specific root length, root surface area, root volume per plant and root morphological parameters of main and lateral roots. These four root parameters per plant of the ME increased up to 4 mmol L^-1^ beyond which notable reduction was observed, indicating exorbitant P concentration has negative effects on root system initiation and the reduction of total root length, surface area and volume seems to be the results of P toxicity. In the case of the NME, these parameters showed continued decrease and were all lower than those of the ME. Our results are in accordance with previous studies that reported higher P efficiency genotypes altered root morphology resulting in greater total root length, root surface area, root volume, and then assimilated greater P (Yang et al., 2010; Fita et al., 2011). Root length and root surface area are deemed to play important roles in the excavation and utilization of soil P (Vance et al., 2003). Zhang et al. (2013a) also reported that seedlings with a higher total root length and root surface area assimilated a larger total amount of P, and accumulated greater DW than that with a lower root surface area. High specific root length is advantageous for seedlings due to a lower resource consumption to yield root length (Pang et al., 2010). In addition, developments of main and lateral roots affect the exploitation volume of the soil resources and assimilation area for the mineral nutrients (Hill et al., 2006; Lynch, 2011). The main root length of *Arabidopsis* increased as response to high P media compared to the relatively low P media (López-Bucio et al., 2005), and the similar pattern was observed in the main root length of the ME which increased up to 4 mmol L^-1^. Lateral root initiation greatly contributes to the spatial configuration of the root system and its allocation is an important factor influencing the average diameter, specific root length of whole root system, and then affects P acquisition from media (Zhu and Lynch, 2004; Pérez-Torres et al., 2008; Liu et al., 2013). Higher allocation of lateral roots were observed in both ecotypes as a result of greater root length than that of main roots, indicating that *P. hydropiper* increased root length mainly through increasing fine root. Root traits such as proliferation and elongation improved plant tolerance for potential element toxicity (White et al., 2013). The inhibition of root growth in tolerant variety or genotype was less serious than sensitive ones under P-deficiency (Zhang et al., 2013b). In our study, the formation of main and lateral roots of the NME, particularly lateral roots, was inhibited by high Po media and the sensitive one (NME) suffered serious high-P toxicity compared to the tolerant one (ME). The ME was able to distribute more lateral roots than the NME to relieve the inhibition of root growth and enhance its tolerance for high Po concentrations as a result of the lateral root length just decreased at levels greater than 4 mmol L^-1^. More allocation of lateral roots was also reported in some P-efficient plants such as maize (Zhu and Lynch, 2004) and *Arabidopsis* (Pérez-Torres et al., 2008). Average diameter of main roots was significantly higher than that of lateral roots in plants with fibrous root system, and lateral root extension required less biomass and phosphorus investment than the extension of main roots, which is helpful to increase specific root length and enhance P uptake (Zhu and Lynch, 2004; Lambers et al., 2006; White et al., 2013). In this study, average diameter of lateral roots was thinner relative to the NME, suggesting that the ME was able to rapidly extend root assimilation area by lessening diameter as result of higher specific root length, greater lateral root length, and surface area. The altered root allocation and preferential root growth in the ME provided the basic conditions to enhance P uptake from high Po media. Therefore, the data (root morphology parameters, biomass, and P content) combined with the above reports suggested that fine root morphology in the ME is the result of greater Po tolerance and it might be an adaptation that contributes to higher P uptake for the ME from phytate.

Another root physiological adaptation is exudation of Apase and phytase to mineralize Po. In the case of Apase, the ME demonstrated the highest enzyme activity when grown under 4 mmol L^-1^ while the NME reached its maximum at 2 mmol L^-1^, which is similar to the pattern of shoot biomass and shoot P accumulation (**Figure 5A**). Gulf and Marshall ryegrass (*L. multiflorum*) had greater Apase activity in root extracts when grown in phytate-sufficient agar substrate (Sharma and Sahi, 2011). Both ecotypes of *P. hydropiper* grown in Po media produced more Apase activity compared with seedlings grown in high Pi soils (Huang et al., 2012), high Pi hydroponic media, livestock wastewater (Ye et al., 2014b), and swine manure soils (Ye et al., 2014a). Our results indicated that phytate-sufficient conditions enhanced the activities of Apase in the two ecotypes of *P. hydropiper*. Phytase activity in both ecotypes of *P. hydropiper* increased with the increasing applications of phytate in the media (**Figure 5B**). This observation agreed with previous studies which reported that P-sufficient enhanced root phytase (Priya and Sahi, 2009; Sharma and Sahi, 2011). The results were also in agreement with our earlier studies which showed that both ecotypes of *P. hydropiper* grown in P-sufficient soils demonstrated greater root phytase activities relative to P-deficient seedlings (Ye et al., 2014a). In the previous reports, phytase activities in several plants were small in proportion to Apase activities (Richardson et al., 2000; Huang et al., 2012; Ye et al., 2014a), which is also noticed in our current study. Phytase demonstrates high affinity for phytate, however, the low solubility of phytate and deficient phytase activity impose restrictions on this mineralization process (Sharma and Sahi, 2005). The wild type of *Arabidopsis* was limited to utilize phytate, whereas transgenic phytase gene from *Aspergillus niger* lines of *Arabidopsis* accumulated more than 10-fold shoot P than the wild type, which attributed great P assimilation to significant increase in total root phytase activity (Richardson et al., 2001). An enhanced phytase activity was observed by inoculation of *Bacillus* isolates, which might be responsible for the high P mobilization and uptake in soybean (Ramesh et al., 2011). In addition, our data, consistent with previous observations on *P. hydropiper*, indicated that the ME showed higher Apase and phytase activities compared to the NME. Similar with our previous researches (Huang et al., 2012; Ye et al., 2014a), great levels of root Apase and phytase activities in *P. hydropiper* enhanced P uptake efficiency by mineralizing Po from phytate media to release available Pi for plant growth. Changes of physiological and biochemical parameters involved in P assimilation from Po culture media are associated with intrinsic gene expression. Recent studies indicated that transgenic expression of APase gene or phytase gene (two *Medicago truncatula* genes) were responsible for great DW performance and P accumulation when transgenic alfalfa (*M. sativa*) were grown on sand culture containing phytate (Ma et al., 2012). Wang et al. (2009) reported that over-expressed *AtPAP15* in soybean significantly increased DW and P contents compared with the wild type when they were grown on sand supplied with phytate. Kong et al. (2014) found that over-expressing purple Apase gene *GmPAP4* enhanced *Arabidopsis* growth and utilization of Po from phytate. Therefore, the molecular mechanisms on P assimilation in both ecotypes of *P. hydropiper* are deserving of further detailed investigation to clarify the complex cause for P enrichment from Po.

## CONCLUSION

In the two ecotypes of *P. hydropiper*, the ME seems to be more tolerant to various concentrations of Po (phytate) and therefore exhibited greater biomass, tissue P content, BCF compared to the NME. A difference in high Po tolerance between the ME and NME caused the variation in root physiological adaptations. The ME showed greater total root length, specific root length, root surface area, and root volume compared with the NME, irrespective of phytate-P level in the growth media. Higher allocation of lateral roots in the ME compared to the NME as a result of the ME demonstrated significantly higher root length, surface area, and volume and finer average diameter in lateral roots when grown in high Po media. Higher APase and phytase activities were also observed in the ME. Thus, high P uptake efficiency from phytate in the ME was due to fine root morphology and high levels of root Apase and phytase activities. Therefore, the ME of *P. hydropiper* is relatively efficient in assimilating P from phytate-rich media and has potential for phytoremediation of contaminated area with high Po.

## AUTHOR CONTRIBUTIONS

Daihua Ye and Tingxuan Li carried out the majority of this research work (experiment design, plant cultivation, chemical analysis, statistical analysis, and writing this paper). Zicheng Zheng and Xizhou Zhang participated in collection of seedlings, plant cultivation, and experiment management. Haiying Yu and Guangdeng Chen participated in the statistical analysis process and modified this manuscript.

## Conflict of Interest Statement

The authors declare that the research was conducted in the absence of any commercial or financial relationships that could be construed as a potential conflict of interest.
